# Microbial Species Isolated from Infected Wounds and Antimicrobial Resistance Analysis: Data Emerging from a Three-Years Retrospective Study

**DOI:** 10.3390/antibiotics10101162

**Published:** 2021-09-24

**Authors:** Valentina Puca, Roberta Zita Marulli, Rossella Grande, Irene Vitale, Antonietta Niro, Gina Molinaro, Silvia Prezioso, Raffaella Muraro, Pamela Di Giovanni

**Affiliations:** 1 Department of Pharmacy, “G. d’Annunzio” University of Chieti-Pescara, 66100 Chieti, Italy; valentina.puca@unich.it (V.P.); irene.vitale@unich.it (I.V.); silvia.prezioso@studenti.unich.it (S.P.); pamela.digiovanni@unich.it (P.D.G.); 2 Center for Advanced Studies and Technology (CAST), “G. d’Annunzio” University of Chieti-Pescara, 66100 Chieti, Italy; 3 Operative Unit of Clinical Pathology, S. Pio Hospital, Vasto, 66054 Chieti, Italy; robymaru@gmail.com (R.Z.M.); antonietta.niro@virgilio.it (A.N.); ginamolinaro87@gmail.com (G.M.); 4 Department of Innovative Technologies in Medicine and Dentistry, “G. d’Annunzio” University of Chieti-Pescara, 66100 Chieti, Italy; raffaella.muraro@unich.it

**Keywords:** antimicrobial resistance, infected wounds, antimicrobial activity, biofilm, co-infections, antibiotics

## Abstract

The antimicrobial resistance is a topic of global interest in the treatment of wound infections. The goal of this retrospective study was both the identification of the microorganisms responsible for wound infections and the determination of their drug susceptibility pattern. The study was performed from 2017 to 2019 and included 239 patients. Thirty-four species were isolated by culture methods and identified and analysed for their susceptibility patterns to antimicrobials through the Walk Away automated system. The presence of one species was the most frequent condition (75.3%), whereas a co-infection was detected in 24.7% of samples. The most common species were Gram-negative (57.9%), amongst which the most prevalent were *Pseudomonas aeruginosa* (40.2%), *Escherichia coli* (20.7%), *Proteus mirabilis* (11.2%), and *Acinetobacter baumannii/haemolyticus* (9.5%). Gram-positive bacteria were observed in 36.6%, *Staphylococcus aureus* (79.4%) being the most predominant species. At least one resistance to antibiotics was detected in 88.2% of isolates, while a multi-drug-resistance versus no less than 6 antimicrobials was detected in 29.2% of isolates. Although multi-drug resistant species and co-infections were observed, those were less frequently observed at the wound site. These conditions make the microorganisms eradication more difficult. The detection of a polymicrobial infection and multi-drug resistant microorganisms followed by a proper therapeutic treatment would lead to the resolution of the infection, promoting wound healing and the limitation of the spread of antibiotic resistance.

## 1. Introduction

The skin represents a defence barrier against the colonization of pathogens. Therefore, the disruption of the normal anatomical structure by surgical operations or by chemical, physical, mechanical and thermal events, with an alteration of skin functions, results in a wound [1]. Skin is exposed to injuries, scratches and it is in contact with the external environment, thus it is more susceptible to colonization by pathogens [2,3,4].

Wounds are divided into two categories: acute and chronic. Acute wounds, like cuts, burns, abrasions and surgical wounds, heal through the regular phases of wound repair and they are caused by external factors. An infected wound affects the quality of life, and compromises the wound’s healing rate [5]. Wound infections represent one third of nosocomial infections among surgical patients and are responsible of 70–80% of mortality [5,6,7]. Wound infections are associated with morbidity and mortality in patients, especially in developing countries, regardless by the type of wound [5,6,8]. Failure in the treatment implies an increase in the healthcare costs, since they involve a prolonged hospitalization due to diagnostic tests, a huge administration of antibiotics and, sometimes, invasive surgery [9].

The diagnosis of infection requires a long time, adequate instrumentation and qualified professionals [10,11] and it is usually based on wound examination, infection biomarker detection, and microbiological analysis. Antibiotic treatment and wound care represent two critical factors for the management of the infection [12]. The antibiotics administration is sometimes empirical, without the support of microbiological analysis [13,14]. The detection of microbial species, pathogens distribution and antimicrobial susceptibility patterns are important aspects, often underestimated, in order to limit the spread of antibiotic-resistant isolates. In particular, the detection of the different microbial species colonizing a wound, as well as their susceptibility to the antimicrobials, can provide an indication for a more appropriate therapy to be administered to patients, significantly reducing the health care costs [15].

On the other side, chronic wounds, like arterial or leg ulcers, take a longer time to heal and they are caused by internal factors that can be associated with diseases like diabetes or immune deficiency diseases [4,10].

A lot of pathogens, like bacteria, viruses and fungal parasites, can be responsible for skin infections, since they find a suitable environment for their colonization and proliferation in the deeper tissues of the skin [4,10,11]. The most common bacterial species that cause wound infections are *Pseudomonas aeruginosa*, *Staphylococcus aureus*, *Klebsiella pneumoniae*, *Enterococcus faecalis* and *Acinetobacter baumannii*. In particular, in the initial phase of infections, within the first week, Gram-positive bacteria, especially *S. aureus,* appear to be the most frequent colonizers [16,17]. From the beginning of the second week, Gram-negative bacteria, such as *P. aeruginosa* and *A. baumannii*, start to colonize the wound, provoking sepsis if they enter the lymphatic system and blood vessels. Awareness has been gained over the last decade on when wound chronicity has been linked to the development of microbial biofilm [1]. It’s well documented, that microorganisms, under stressful conditions, cooperate and communicate with each other, sharing the same biological niche or body district, guaranteeing their mutual survival [18,19]. The EPS biofilm matrix protects microorganisms from the action of antimicrobial drugs, as well as the host immune system. Thus, microorganisms involved in co-infections can develop polymicrobial biofilms characterized, not only by intrinsic genotypic resistances, but also by a phenotypic resistance or antimicrobial drugs tolerance associated with biofilm matrix [20]. We have to consider that the antimicrobial susceptibility pattern, in routine analysis, is determined on the planktonic phenotype of a single isolate. However, when the microbial cells are aggregated within a biofilm, antimicrobial concentrations up to 4 times the Minimal Inhibitory Concentration (MIC), are required for biofilm eradication [20,21]. The biofilm represents one of the most complicated factors implicated in wounds healing, with a predominance rate of 60% and 100% in chronic wounds. These types of wounds are difficult to treat and they constitute a significative challenge both in military medical centers and public healthcare facilities [22].

The infections associated with biofilms are debilitating for patients since they can persist for months causing patients to lose hope of recovery. In particular, biofilm has been detected in chronic leg ulcers [23,24], diabetic food ulcers [23], pressure ulcers [25], burns [26], malignants wounds [27] and surgical wounds [28]. The biofilm represents a suitable niche for the exchange of resistance genes, therefore, chronic patients might represent, as previously mention by Howell-Jones et al., a “high-risk group for the acquisition, carriage and dissemination of antibiotic-resistant microorganisms” [29].

The World Health Organization (WHO) has considered the antimicrobial resistance as top ten threats to global health and is working to increase the knowledge in this field, in order to both decrease the rate of microbial infections and to provide a more aware and appropriate use of antimicrobial drugs [30]. Drug resistance is due to the inappropriate use of antimicrobials in humans and animals, and the onset of the so-called “superbugs” or multi-drug resistant strains, represents a public health concern [5]. The WHO report of 2014 indicates that multi-drug resistant pathogens are responsible for about 25,000 death and 23,000 death every year in Europe and the United States, respectively. Moreover, about the 50% of infections associated with *E*. *coli*, *K*. *pneumoniae*, *S*. *aureus* and *P*. *aeruginosa* showed resistance against the most effective antimicrobials such as third-generation cephalosporin [5,31].

In fact, confirming the above, among the best known antibiotic resistance microorganisms discovered in hospitals, Rice et al., set up the so called “ESKAPE” cluster composed by *Enterococcus faecium*, *S. aureus*, *K. pneumoniae*, *A. baumannii*, *P. aeruginosa*, and *Enterobacter* species [32]. Among these, the most dangerous ones are methicillin-resistant *S. aureus*, vancomycin-resistant *S.aureus*, carbapenem-resistant *Acinetobacter* species, quinolones and carbapenems-resistant *P. aeruginosa* [33,34]. 

The consequences of antibiotic resistance are very serious and they lead to financial burdens. A meta-analysis showed that in the United States, the treatment of hospital-acquired infections costs almost $10 billion annually [35]. On the basis of these concerns, the aim of the present retrospective study was the identification of the microbial species responsible for wound infections, the determination of possible co-infection conditions as well as the analysis of the drugs susceptibility pattern associated. The reported data might represent an indication for clinicians in order to improve the surveillance, the prevention and the control of wound infections.

## 2. Results

Overall in this research paper, 239 wound samples were collected from 239 patients having a median age of 73 years (interquartile range: 55–84 years), collected from 2017 to 2019 (Table 1). 

The antibiotic susceptibility pattern evaluated showed the presence of at least one resistance in the 88.2% of wound samples (Table 2). In particular, 12.2% of the samples with at least one resistance showed resistance versus only one tested antibiotic, 20.8% showed resistance versus two antibiotics, 11.6% showed resistance toward 3 antibiotics, 14.6% showed resistance to 4 antibiotics, 11.6% showed resistance to 5 antibiotics and, surprisingly, 29.2% showed a resistance pattern toward at least 6 antibiotics tested. The analysis of the distribution of the resistances, divided into survey’s year, showed an increase in the number of the resistances from 2017 to 2019 (Table 2). 

The microbiological analysis detected 34 species as reported in Table 3 and the most common bacterial species identified were Gram-negative (57.9%) in respect to Gram-postive (36.6%). Among Gram-negative microorganisms, *P. aeruginosa* (40.2%), *Escherichia coli* (20.7%), *Proteus mirabilis* (11.2%) and *A. baumannii/haemolyticus* (9.5%), were the most represented species while, among Gram-positive bacteria, *S. aureus* rapresents the predominant species in the 79.4% of the cases, followed by *Staphylococcus haemolyticus* (4.4%) and *Enterococcus faecalis* and *Staphylococcus epidermidis* (2.7% in both cases). For what concerns the presence of fungi, these last ones were detected just in the 5.5% of swabs, highlighting most frequently the presence of *Candida albicans* (41.2%) and *Candida glabrata* and *parapsilosis* (17.6% in both cases). Regarding the distribution of microorganisms stratified by years, an increase of the frequency of isolation of *P. aeruginosa* as well as *A*. *baumannii*, *E. coli* and *P. mirabilis*, was observed from 2017 to 2019. On the contrary, the distribution of *S. aureus* and *E.* *cloacae* remained constant over time. Interestingly, *K. pneumoniae*, *M*. *morganii*, *S*. *marcescens* and *C. albicans* showed an increase in the distribution in 2018 followed by a decrease in 2019 (Table 3).

One hundred forty-three participants came from community-based settings (59.8%) and the most common bacterial species identified at the wound site were *S. aureus* (38.5%) and *P. aeruginosa* (29.4%). The hospital-based participants were 96 (40.2%) and *S. aureus* (36.5%), *E. coli* (15.6%) and *P. aeruginosa* (13.5%) were the bacteria most frequently found in the samples.

**Figure 1:**
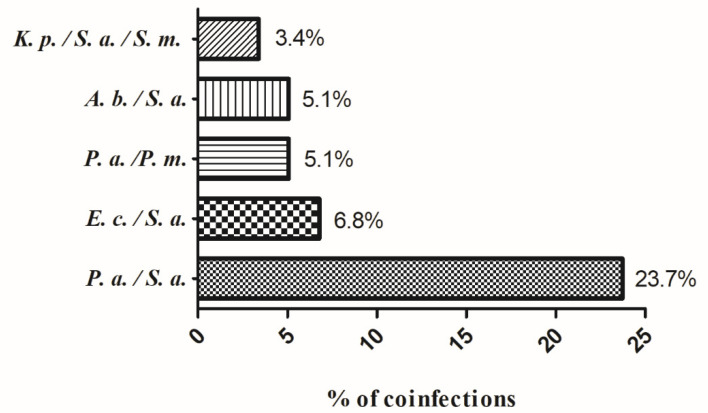
Percentage of co-infections detected in samples with multiple species. The percentage of co-infections was calculated by considering the total number of the co-infections. *A. b*.: *Acinetobacter baumannii/haemolyticus*; *E. c*.: *Escherichia coli*; *K. p*.: *Klebsiella pneumoniae*; *P. a*.: *Pseudomonas aeruginosa*; *P. m*.: *Proteus mirabilis*; *S. a*.: *Staphylococcus aureus*; *S. m*.: *Serratia marcescens*.

The presence of only one species was the most frequent condition and it was found in 180 samples corresponding to 75.3% of infected wounds, instead of co-infection by different species which was found in 59 samples, corresponding to 24.7%. The species more frequently identified in a co-infection condition were *P. aeruginosa* and *S. aureus* (23.7%,) followed by *E. coli* and *S. aureus* (6.8%), *P.mirabilis* and *P. aeruginosa* (5.1%), *A. baumannii/haemolyticus* and *S. aureus* (5.1%) (Figure 1). Only a triple co-infection was identified and involves *K. pneumoniae, S. aureus* and *S. marcescens* with a percentage of 3.4%. Among the co-infections, 39% derived from chronic wounds.

We didn’t find a significant difference in the number of bacteria when we compared the age of the patients (younger vs. older median age, *p* = 0.605) and the gender (*p =* 0.956) (Table 4). 

Among multi-resistant microorganisms, *P. mirabilis* is the one that most frequently presents a number of resistances equal to or greater than 6 (21.0%), followed by *S. aureus* (19.4%), *P. aeruginosa* and *E. coli* (12.9% in both cases), as shown in Table 5.

There was one isolate from four different species that showed a resistance versus 12 antimicrobials: *C. freundii*, *E. coli*, *K. pneumoniae* and *S. aureus,* while one *P. mirabilis* isolate was resistant toward 10 antimicrobials (Table 6).

We didn’t find a statically significant difference in microbial resistance when we compared the age of the patients (younger vs. older median age, *p* = 0.080) and the gender (*p* = 0.625) (Table 7).

The percentage of resistance towards antimicrobials has been detected and ampicillin was the antimicrobial toward which the isolated strains showed the higher percentage of resistance corresponding to 56.1% (134 isolates), followed by Gentamicin 32.2 (77 isolates) Penicillin 27.6% (66 isolates), Trimethoprim/Sulfamethoxazole 25.9% (62 isolates), and Piperacillin 24.3% (58 isolates) (Tables S1 and S2). 

Regarding the most effective antimicrobials, the analysis of the susceptibility pattern to antimicrobial drugs showed that the antimicrobials most active against Gram-negative bacteria were amikacin and imipenem. In particular, amikacin worked well against all Gram-negative species except *A. baumannii* in which 12 resistant isolates were detected out of 17 tested strains; imipenem was active against all Gram-negative species except *P. aeruginosa* in which 6 resistant isolates were detected out of 72 tested strains (Table S1). 

On the other side the antimicrobials most active towards Gram-positive bacteria were found to be vancomycin and trimethoprim/sulfamethoxazole (Table S2). Vancomycin has been shown to be active against all Gram-positive species tested on the basis of EUCAST guidelines; finally, trimethoprim/sulfamethoxazole worked well against all Gram-negative species on the basis of EUCAST guidelines (in addition, 8 resistant strains out of 90 tested *S. aureus* isolates have been detected).

**Figure 2:**
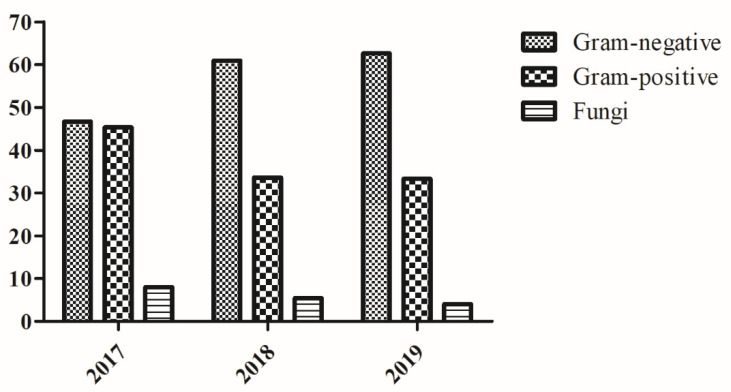
Percentage distribution of groups of microorganisms stratified by year.

The analysis of the distribution of the groups of microorganisms, divided into survey’s year, showed an increase in the frequency of Gram-negative bacteria over time, while the frequency of Gram-positive bacteria decreases slightly over the years. Thus, in 2017 the frequency of Gram-positive and Gram-negative bacteria is almost comparable, while in 2018 and 2019 a major representativeness of Gram-negative bacteria was registered (Figure 2). A statistically significant rising linear trend was observed for Gram-negative bacteria (*p* = 0.031) in the study period.

Stratifying the data by source of the samples (community or hospital), the rise linear trend for Gram-negative bacteria, in the study period, remains statistically significant both for the samples coming from the hospital (*p* = 0.031) and for those coming from the community (*p* = 0.027).

## 3. Discussion

Routine testing in microbiology laboratories has largely based on culture methods that allow to detect and isolate potential pathogens from swabs, pus or tissue biopsies in order to identify the species and to determine antimicrobials susceptibilities as a guide for a therapeuthic treatment. Standardized methodology enables international surveillance to limit the spread of the antibiotic resistance [1]. In the present retrospective study, 34 microbial species were isolated from wounds with signs of infection. Gram-negative bacteria were more represented (57.9%) in respect to Gram-positives (36.6%), confirming the results reported in other studies [36,37,38]. The majority of the wounds were colonized with a single bacterial species. These data are confirmed by Mohammed et al., that found single bacterial growth in the 81.7% of the swab cultures [39], while Yeong et al. showed a higher number of polymicrobial resistant species in wound bacterial cultures within the first 72 h [40]. The most common isolate microorganism was *S. aureus*, followed by *P. aeruginosa* and *E. coli*, as previously demonstrated by other authors [36]. These bacterial species induce a damage on wound healing [41,42].

For what concerns polymicrobial infections, the most frequent associated microorganisms were *S. aureus* and *P. aeruginosa,* in accordance with the data reported in other studies that showed that *S. aureus* and *P. aeruginosa* are the predominant species in wound infections [16,17]. However, *S. aureus* has been detected associated also with *E. coli* in (6.8%) and *A. baumannii* in (5.1%). In the same way, *P. aeruginosa* has been detected associated also with *P. mirabilis* (5.1%). In 3.4% a co-infection of 3 different bacterial species corresponding to *K. pneumoniae* plus *S. aureus* plus *S. marcescens* has been observed. The presence of polymicrobial infections makes the eradication of microorganisms more difficult. The polymicrobial nature of chronic wounds is likely to provide a suitable environment for the horizontal gene transfer between microorganisms [29].

In the present study we detected 29.2% of microbial strains which showed a resistance against at least 6 antimicrobial agents, in particular the species involved were *S. aureus* and *P. aeruginosa* which represent the most common isolated species, followed by *E. coli*, *K. pneumoniae*, *P. mirabilis* and *A. baumannii*. These species have been also found in a co-infection condition. Therefore, in addition to being characterized by a genotypic multi-drug resistance, the capability of these microorganisms to produce a polymicrobial biofilm makes the therapeutic treatment more difficult. The presence of multi-drug resistant strains, the variability of the biofilm composition, its tolerance towards the antibiotics, as well as the possible polymicrobial nature of biofilms, suggest the need for multi-target or combinational approaches [8,20,43]. Among the multi-drug resistant strains we found some even resistant to 12 different antimicrobials belonging to *Citrobacter freundii*, *E. coli*, *K. pneumoniae*, *S. aureus.* The results obtained showed a high resistance rates, of the species isolated, to ampicillin (56.1%), gentamicin (32.2%), penicillin (27.6%), trimethoprim/sulfamethoxazole (25.9%), and piperacillin (24.3%) [36,41,44]. Unlike most of the studies in literature, ciprofloxacin was not detected among the most resistant antibiotics [36,41,45,46,47,48]. Amikacin worked well against all Gram-negative species except *A. baumannii* and imipenem was effective against all Gram-negative species except *P. aeruginosa*. These data are partially confirmed by other studies [36,47] which showed that Gram-negative bacteria have low resistance rates toward amikacin (3.6%); however, Guan et al. found that that *P. aeruginosa*, *P. mirabilis* and *P. vulgaris* were highly resistant to imipenem (80%–100%) [36]. Surprisingly, we found that *M. morganii* was sensitive to imipenem, while in the work of Guan et al., this bacterial species showed a resistance rate of 80% [36]. The resistance rates of *A. baumanii* and *P. aeruginosa*, identified in our samples, were enough different when compared to the data in literature. *A. baumanii* was resistant to amikacin in 70.6%, whereas Guan et al. demonstrated that it was resistant for 13.3%. *A. baumanii* was 100% sensitive to imipenem, while in the work of Li et al. it was demonstrated a resistance rate of 83.3% [44]. Furthermore, Li et al. showed that *P. aeruginosa* was 97.5% resistant to trimethoprim/sulfamethoxazole, while our data dysplayed a resistance rate of 1.4% [44].

On the contrary, the antimicrobial drugs that were found to be most active were amikacin and imipenem against Gram-negative species and vancomycin and trimethoprim/sulfamethoxazole versus Gram-positives. The efficacy of amikacin, vancomycin and imipenem was reported also in other studies [15]. Several studies, reported in literature, confirmed our results demonstrating that vancomycin appears to be the most effective antibiotic towards Gram-positive bacteria [36,41,44,49]. In addition, we investigated the sensitivity to amikacin and imipenem which were very effective. In particular, imipenem, unlike amikacin, was not considered in other studies [49,50]. On the other hand, Wong et al. found that amikacin and meropenem were the most effective antibiotics against Gram-negative bacteria in chronic wounds [41].

Another important aspect is related to the stratification of the isolates by year. In particular, while in 2017 the percentage of isolated Gram-positive and Gram-negative bacteria was almost identical, in the following years, 2018 and 2019, there was an increase of isolation, equal to about 60% of Gram-negative bacteria and a slight decrease in the detection of Gram-positive bacteria. On the contrary, the percentage of fungal strains has no undergone great variations over time suggesting a greater spread/transmission of Gram-negative species. The present study, however, presents some limitations, in particular (1) the presence of anaerobic and microaerophilic microorganisms was not detected; (2) in the same way, it was not detected the possible presence of biofilm as well as the presence of microorganisms in the VBNC state, particularly in the co-infection conditions; (3) we have no information about any pathologies of the patients considered in the study as well as any therapies performed because the samples were collected for diagnostic purposes independently of the study (information bias), finally, we analyzed the data over three years provided by a single hospital, therefore, further studies will be necessary to analyze the spread of multi-drug resistant species in a larger area of the region.

## 4. Conclusions

Wound infections are underestimated problems that result into a chronic disease. The data reported in the present study might help clinicians to establish, on the basis of microbiological analysis, guidelines for the choice of an appropriate therapeutic regimen, in order to both treat wound infections and limit the development of multi-drug resistant strains.

In particular, the increase in drug-resistant Gram-negative microorganisms over the three-year period suggests the need for careful and up-to-date monitoring of multidrug-resistant strains diffusion in the various geographical areas of the country. The identification of the most effective antibiotics against some microbial species could orient the clinicians towards the administration of some antimicrobials rather than others, resulting in a limitation in the use of less effective drugs for the treatment of wound infections. In addition, the presence of multidrug-resistant strains even to 12 different drugs should make us reflect on the importance of both a correct use of antibiotics, avoiding the prescription of empirical therapies, and the development of biofilms. The development of standardized in vitro methods to determine not just the antimicrobial activity versus microorganisms in the planktonic phenotype, but also the effectiveness versus bacteria in the biofilm phenotype, lead both to the resolution of the infection and to the wound healing. A polymicrobial biofilm is more difficult to eradicate, therefore, more attention should be paid to the study of this aspect by also evaluating additional microbiological analyses aimed at evaluating the Minimal Biofilm Eradication Concentration in order to find the most effective therapy for biofilm eradication to avoid relapses. Another important aspect to take into consideration is the detection of anaerobic microorganisms as well as of viable but non-culturable bacteria. The molecular technique demonstrated that the majority of wounds are polymicrobial [51,52], suggesting that the microbial composition inside a wound is very complex. In fact, although it has been shown in the past that most of the microorganisms detected in infected wounds are aerobic, recently it has been demonstrated that anaerobic microorganisms are a representative component of the wound microbiota, especially, in chronic wounds [52,53,54]. Therefore, a more in-depth studies of all the microbial species present within an infected wound is necessary in order to better understand the interactions between the different microorganisms and their ability to cooperate with each other. Such information would help us to select the most effective drugs for the treatment and resolution of infected wounds. Finally, it might be important to evaluate, inside a polymicrobial infection, the concentration of the different species to establish if there is a prevalence of one species over another or if there is a balance between the different species.

Limiting the spread of antibiotic resistance is a public health goal, also promoted and supported by the WHO, to be pursued and requires the control of multi-resistant bacteria and the availability of revised therapeutic regimens.

## 5. Material and Methods

### 5.1. Patients

In the present retrospective study a total of 239 samples were collected from infected wounds. The samples were taken from patients, 54% of female gender and 46% of male gender between 7 and 98 years of age, with a median age of 73 years, in the three-year period from 1st January 2017 to 31st December 2019. Samples collection was carried out by nurses on the basis of an established and detailed internal protocol provided by the S. Pio Hospital. The biological samples examines came from both hospitalized patients (40.2%) and individuals who accessed the hospital for later sample analysis at the request of their medical doctor (59.8%). The microorganisms were isolated from other infected wounds (21.7%), fistula (10.5%), ulcers (57.3%) and abscesses (10.5%). Among the other infected wounds, the 4.2% were surgical wounds. The study and the analysis were carried out at the Laboratory of Microbiology, Operative Unit of Clinical Pathology of “S. Pio” Hospital of Vasto, Chieti, Italy.

### 5.2. Sample Collection

Before sample collection, the cleaning of the edges of the wound and removal of superficial exudate by washing with physiological sterile solution was carried out. This kind of operation is important to remove environmental microorganisms colonizing wound surface. The sampling collection was performed on the basis of the type of wound. In particular, the sampling trought the use of the swab has been carried out in the case in which the wound was superficial and the sample has been taken from the lesion by rotating the swab in a “Z” movement, avoiding touching the surrounding skin. Finally the swab was reinserted into the appropriate tube containing the Amies transport medium (MicroBiotech, Maglie, Italy). Amies Transport Medium is a non-nutritive semi-solid medium used for transport samples to be subjected to microbiological analysis. On the contrary, sampling by aspiration has proved more appropriate in case of deep lesions or abscess.

The operator, after injecting the physiological solution on the wound, aspirates with a siringe sterile needle at least 0.5–1 mL of material at the deepest part of the lesion and transfers it into a blood culture medium. Once collected, the samples were transported to the microbiology laboratory and housed in the analytical system of microbial detection BacT/ALERT 3D (BioMérieux, Marcy-l’Etoile, France) which monitors bacterial growth. The bottle must be loaded promptly, to prevent microbial growth reaches the plateau, a condition that would create problems in the detection of the microorganisms by the instrument. The samples have been then monitored continuously, 24 h a day for the determination of the growth curve. Monitoring of incubating vials is continuous and readings are taken every 10 minutes until the result is formulated. The incubation temperature is 35 °C and the incubation times are 7 days. After 7 days of incubation, if the bottle failed, the system monitoring automatically detected the failure.

### 5.3. Species Identification and Antimicrobial Susceptibility Pattern Determination

The positive samples incubated in BacT/ALERT 3D were then plated on Mannitol Salt agar, Mc Conkey agar, Sabourad agar and blood agar (Biomerieux, Marcy-l’Étoile, France). The plates were then incubated at 37 °C in aerobic conditions for 18–24 h except for Sabourad agar plates that were incubated at 30 °C in aerobic conditions for 48 h. The identification of the microorganisms was based on the analysis of both colonies morphology and characteristics and the use of MicroScan WalkAway system (Beckman Coulter, Brea, CA, USA) which allow both the identification of the microbial species by using biochemical reactions and the determination of the antimicrobial susceptibility pattern by using specific panels (Beckman Coulter, Brea, CA, USA). In particular, Pos Breakpoint combo Panel type 32 (PBC 32) have been used both in the identification of aerobic and facultative fast-growing Gram-positive cocci, some fastidious aerobic Gram-positive cocci and *Listeria monocytogenes* and for the determination of susceptibility to 30 antimicrobials agents, among which, for each microorganism, only those indicated by EUCAST guidelines are reported [55]. Neg Breakpoint Combo Panel 45 and 46 (NBC 45, NBC 46) have been used in the identification at the species level of aerobic and facultative aerobic Gram-negative bacilli and for the determination of antimicrobial susceptibility against 27 antimicrobials, also in this case, only those indicated by EUCAST guidelines are reported [55]. MICroSTREP plus panels have been used for the identification of aerobic streptococci included *S. pneumoniae*, and the determination of the antimicrobial susceptibility versus 27 antimicrobial agents. Finally, for fungi, has been used Rapid Yeast ID panel which allows the rapid identification of yeasts. For the analyzed samples the guidelines do not provide for the execution of the antifungal susceptibility test since the therapy is standardized and is chosen by the clinician. All the experimental procedures followed the manufacture’s indications.

### 5.4. Statistical Analysis

The categorical variables are expressed as number and percentage and the continuous variables as median and interquartile range (IQ). The Mantel-Haenzsel chi-square test for trend was used to assess whether there was a statistically significant linear trend in the proportion of infections due to the most frequent microorganisms over the study period. For analyses, a *p*-value ≤ 0.05 was considered statistically significant. Statistical analyses were performed using IBM^®^ SPSS Statistics v 20.0 software (SPSS Inc., Chicago, IL, USA) and GraphPad PrismSoftware, version 7 (GraphPad Software, La Jolla, CA, USA).

### 5.5. Ethical Considerations

Ethical approval was not required because the study was retrospective and used data encrypted by Operative Unit of Clinical Pathology before the analysis. In this way, it was not possible to trace the subjects’ identity. The study was conducted in conformity with the Italian Law on privacy (Art. 20–21 DL 196/2003) published on the Official Journal n.190 of 14 August 2004).

## Figures and Tables

**Table 1:** Characteristics of the sample stratified by survey’s years.

Characteristics	2017 N (%)	2018 N (%)	2019 N (%)	Total N (%)
Sample	62 (25.9)	81 (33.9)	96 (40.2)	239 (100.0)
Gender				
F	41 (31.8)	47 (36.4)	41 (31.8)	129 (54.0)
M	21 (19.1)	34 (30.9)	55 (50.0)	110 (46.0)
Age, years median (IQ)	70.5 (51–80)	75 (58.5–86)	73 (55–84)	73 (55–84)
Setting				
community-based	34 (23.8)	54 (37.8)	55 (38.4)	143 (59.8)
hospital-based	28 (29.2)	27 (28.1)	41 (42.7)	96 (40.2)
Co-infection				
no	50 (27.8)	59 (32.8)	71 (39.4)	180 (75.3)
yes	12 (20.3)	22 (37.3)	25 (42.4)	59 (24.7)

**Table 2:** Resistance profile stratified by survey’s year.

Resistance Profile	2017 N (%)	2018 N (%)	2019 N (%)	Total N (%)
Resistance				
no	8 (28.6)	11 (39.3)	9 (32.1)	28 (11.8)
yes	54 (25.6)	70 (33.2)	87 (41.2)	211 (88.2)
Multi-resistance				
no	7 (26.9)	5 (19.2)	14 (53.9)	26 (12.2)
2 antimicrobials	17 (38.6)	14 (31.8)	13 (29.6)	44 (20.8)
3 antimicrobials	3 (12.5)	10 (41.7)	11 (45.8)	24 (11.6)
4 antimicrobials	5 (16.1)	11 (35.5)	15 (48.4)	31 (14.6)
5 antimicrobials	5 (20.8)	6 (25.0)	13 (54.2)	24 (11.6)
≥6 antimicrobials	17 (27.4)	24 (38.7)	21 (33.9)	62 (29.2)

**Table 3:** Distribution of microorganisms stratified by group and survey’s year.

Species	2017 N (%)	2018 N (%)	2019 N (%)	Total N (%)
Gram-negative	40 (22.3)	63 (35.2)	76 (42.5)	179 (57.9)
*Acinetobacter baumannii/haemolyticus*	4 (23.6)	5 (29.4)	8 (47.0)	17 (9.5)
*Alcaligenes* sp.	3 (100.0)	-	-	3 (1.7)
*Citrobacter freundii*	1 (50.0)	-	1 (50.0)	2 (1.1)
*Enterobacter cloacae*	2 (33.3)	2 (33.3)	2 (33.3)	6 (3.4)
*Escherichia coli*	9 (24.4)	11 (29.7)	17 (45.9)	37 (20.7)
*Klebsiella ornithinolytica*	-	-	1 (100.0)	1 (0.6)
*Klebsiella pneumoniae*	1 (20.0)	3 (60.0)	1 (20.0)	5 (2.8)
*Morganella morganii*	-	3 (60.0)	2 (40.0)	5 (2.8)
*Proteus mirabilis*	5 (25.0)	6 (30.0)	9 (45.0)	20 (11.2)
*Proteus vulgaris*	-	1 (100.0)	-	1 (0.6)
*Providencia* sp.	-	2 (100.0)	-	2 (1.1)
*Pseudomonas aeruginosa*	14 (19.4)	25 (34.8)	33 (45.8)	72 (40.2)
*Pseudomonas fluorescens/putida*	-	1 (100.0)	-	1 (0.6)
*Serratia marcescens*	1 (14.3)	4 (57.1)	2 (28.6)	7 (3.9)
Gram-positive	40 (35.5)	37 (32.7)	36 (31.8)	113 (36.6)
*Enterococcus avium*	1 (100.0)	-	-	1 (0.9)
*Enterococcus faecalis*	2 (66.7)	1 (33.3)	-	3 (2.7)
*Staphylococcus aureus*	29 (32.2)	32 (35.6)	29 (32.2)	90 (79.4)
*Staphylococcus auricularis*	1 (50.0)	-	1 (50.0)	2 (1.8)
*Staphylococcus epidermidis*	3 (100.0)	-	-	3 (2.7)
*Staphylococcus haemolyticus*	2 (40.0)	1 (20.0)	2 (40.0)	5 (4.4)
*Staphylococcus lugdunensis*	1 (50.0)	-	1 (50.0)	2 (1.8)
*Staphylococcus schleiferi* subsp. *coagulans*	-	-	1 (100.0)	1 (0.9)
*Staphylococcus sciuri*	-	-	1 (100.0)	1 (0.9)
*Staphylococcus simulans*	1 (50.0)	1 (50.0)	-	2 (1.8)
*Streptococcus agalactiae*	-	-	1 (100.0)	1 (0.9)
*Streptococcus pyogenes*	-	1 (100.0)	-	1 (0.9)
*Streptococcus salivarius*	-	1 (100.0)	-	1 (0.9)
Fungi	8 (47.0)	5 (29.4)	4 (23.6)	17 (5.5)
*Candida albicans*	2 (28.6)	3 (42.8)	2 (28.6)	7 (41.2)
*Candida glabrata*	2 (66.7)	1 (33.3)	-	3 (17.6)
*Candida guilliermondii*	-	-	1 (100.0)	1 (5.9)
*Candida parapsilosis*	2 (66.7)	1 (33.3)	-	3 (17.6)
*Candida stellatoidea*	1 (100.0)	-	-	1 (5.9)
*Candida tropicalis*	-	-	1 (100.0)	1 (5.9)
*Candida* sp.	1 (100.0)	-	-	1 (5.9)

Note: The total number of the microorganisms is greater than the number of samples because co-infection conditions, based on the colonization of different/more species, have been detected.

**Table 4:** Distribution of number of species stratified for age of the patients (younger vs. older median age) and gender.

	Infection			*p*
	1 Species N (%)	2 Species N (%)	3 Species N (%)	
age				
younger median age	83 (46.1)	25 (51.0)	6 (60.0)	0.605
older median age	97 (53.9)	24 (49.0)	4 (40.0)	
gender				
F	97 (53.9)	27 (55.1)	5 (50.0)	0.956
M	83 (46.1)	22 (44.9)	5 (50.0)	

**Table 5:** Distribution of microorganisms with ≥6 resistances.

Microorganisms	N (%)
*Acinetobacter baumannii/haemolyticus*	6 (9.7)
*Citrobacter freundii*	1 (1.6)
*Enterobacter cloacae*	1 (1.6)
*Escherichia coli*	8 (12.9)
*Klebsiella pneumoniae*	4 (6.5)
*Morganella morganii*	2 (3.2)
*Proteus mirabilis*	13 (21.0)
*Pseudomonas aeruginosa*	8 (12.9)
*Staphylococcus aureus*	12 (19.4)
*Staphylococcus auricularis*	2 (3.2)
*Staphylococcus epidermidis*	3 (4.8)
*Staphylococcus haemolyticus*	2 (3.2)

**Table 6:** Distribution of microorganisms stratified by number of resistances.

Microorganisms	N. of Resistances										
	1 N (%)	2 N (%)	3 N (%)	4 N (%)	5 N (%)	6 N (%)	7 N (%)	8 N (%)	9 N (%)	10 N (%)	12 N (%)
*Acinetobacter baumannii/haemolyticus*	1 (3.8)	1 (2.3)	4 (16.7)	2 (6.5)	3 (12.4)	3 (14.3)	3 (17.7)	-	-	-	-
*Citrobacter freundii*	-	1 (2.3)	-	-	-	-	-	-	-	-	1 (25.0)
*Enterobacter cloacae*	-	3 (6.8)	-	2 (6.5)	-	1 (4.8)	-	-	-	-	-
*Entrococcus faecalis*	1 (3.8)	-	-	-	-	-	-	-	-	-	-
*Escherichia coli*	4 (15.5)	3 (6.8)	3 (12.5)	6 (19.3)	4 (16.6)	4 (19.0)	1 (5.8)	1 (11.1)	1 (10.0)	-	1 (25.0)
*Klebsiella ornithinolytica*	-	-	-	-	1 (4.2)	-	-	-	-	-	-
*Klebsiella pneumoniae*	-	-	-	-	1 (4.2)	-	2 (11.8)	-	1 (10.0)	-	1 (25.0)
*Morganella morganii*	-	-	-	2 (6.5)	1 (4.2)	1 (4.8)	1 (5.8)	-	-	-	-
*Proteus mirabilis*	1 (3.8)	2 (4.5)	2 (8.3)	1 (3.2)	1 (4.2)	3 (14.3)	2 (11.8)	2 (22.2)	5 (50.0)	1 (100.0)	-
*Proteus vulgaris*	-	-	-	-	1 (4.2)	-	-	-	-	-	-
*Providencia* sp.	-	1 (2.3)	-	1 (3.2)	-	-	-	-	-	-	-
*Pseudomonas aeruginosa*	12 (46.2)	6 (13.7)	3 (12.5)	3 (9.7)	6 (25.0)	2 (9.5)	5 (29.5)	1 (11.1)	-	-	-
*Serratia marcescens*	1 (3.8)	2 (4.5)	2 (8.3)	1 (3.2)	1 (4.2)	-	-	-	-	-	-
*Staphylococcus aureus*	4 (15.5)	24 (54.5)	6 (25.0)	12 (38.7)	4 (16.6)	5 (23.8)	2 (11.8)	2 (22.2)	2 (20.0)	-	1 (25.0)
*Staphylococcus auricularis*	-	-	-	-	-	-	-	2 (22.2)	-	-	-
*Staphylococcus epidermidis*	-	-	-	-	-	2 (9.5)	-	-	1 (10.0)	-	-
*Staphylococcus haemolyticus*	1 (3.8)	-	1 (4.2)	1 (3.2)	-	-	1 (5.8)	1 (11.1)	-	-	-
*Staphylococcus lugdunensis*	1 (3.8)	1 (2.3)	-	-	-	-	-	-	-	-	-
*Staphylococcus sciuri*	-	-	-	-	1 (4.2)	-	-	-	-	-	-
*Staphylococcus simulans*	-	-	2 (8.3)	-	-	-	-	-	-	-	-
*Streptococcus salivarius*	-	-	1 (4.2)	-	-	-	-	-	-	-	-

**Table 7:** Distribution of microbial resistance stratified for age of the patients (younger vs. older median age) and gender.

	N. of Resistances											*p*
	1 N (%)	2 N (%)	3 N (%)	4 N (%)	5 N (%)	6 N (%)	7 N (%)	8 N (%)	9 N (%)	10 N (%)	12 N (%)	
age												0.080
younger median age	14 (53.8)	29 (65.9)	13 (54.2)	12 (38.7)	8 (33.3)	9 (29.0)	10 (59.8)	5 (55.6)	2 (20.0)	1 (100.0)	1 (25.0)	
older median age	12 (46.2)	15 (34.1)	11 (45.8)	19 (61.3)	16 (66.7)	12 (71.0)	7 (41.2)	4 (44.5)	8 (80.0)	0 (0.0)	3 (75.0)	
gender												0.625
F	14 (53.8)	20 (45.5)	13 (54.2)	17 (54.8)	13 (54.2)	12 (57.1)	11 (64.7)	7 (77.8)	6 (60.0)	1 (100.0)	2 (50.0)	
M	12 (46.2)	24 (54.5)	11 (45.8)	14 (45.2)	11 (45.8)	9 (42.9)	6 (35.3)	2 (22.2)	4 (40.0)	0 (0.0)	2 (50.0)	

## Data Availability

The datasets generated and analyzed in the current study are available from the corresponding author on reasonable request.

## Supplementary Materials

The following are available online at https://www.mdpi.com/article/10.3390/antibiotics10101162/s1, Table S1. Drug resistance patterns of Gram-negative bacteria; Table S2. Drug resistance patterns of Gram-positive bacteria.
