# Supplementary material for: Microbial Species Isolated from Infected Wounds and Antimicrobial Resistance Analysis: Data Emerging from a Three-Years Retrospective Study
Source: Antibiotics (Basel). 2021 Sep 24;10(10):1162. doi: 10.3390/antibiotics10101162 (PMC8532735; doi:10.3390/antibiotics10101162)
Supplement: Supplementary file 1 [file antibiotics-10-01162-s001.zip › antibiotics-1350904-supplementary.pdf]

Supplementary Materials

Microbial species isolated from infected wounds and antimicrobial resistance analysis: Data emerging from a three-years retrospective study

Valentina Puca[1,2§], Roberta Zita Marulli[3,§], Rossella Grande[1,2*], Irene Vitale[1], Antonietta Niro[3], Gina Molinaro[3], Silvia Prezioso[1], Raffaella Muraro[4] and Pamela di Giovanni[1]

[1] Department of Pharmacy, "G. d'Annunzio" University of Chieti-Pescara, 66100 Chieti, Italy; valenti-na.puca@unich.it (V.P.); rossella.grande@unich.it (R.G.); irene.vitale@unich.it (I.V.); sil-via.prezioso@studenti.unich.it (S.P.), pamela.digiovanni@unich.it (P.D.G.).

[2] Center for Advanced Studies and Technology (CAST), "G. d'Annunzio" University of Chieti-Pescara, 66100 Chieti, Italy; valentina.puca@unich.it (V.P.); rossella.grande@unich.it (R.G.)

[3] Operative Unit of Clinical Pathology, S. Pio Hospital, Vasto (CH), Italy; robymaru@gmail.com (R.Z.M.); anto-nietta.niro@virgilio.it (A.N.); ginamolinaro87@gmail.com (G.N)

[4] Department of Innovative Technologies in Medicine and Dentistry, "G. d'Annunzio" University of Chieti-Pescara, 66100 Chieti, Italy; raffaella.muraro@unich.it (R.M.)

§ VP and RZM contributed equally to the work.

* Correspondence: Rossella Grande, Department of Pharmacy, "G. d'Annunzio" University of Chieti-Pescara, 66100 Chieti, Italy; rossella.grande@unich.it

Table S1. Drug resistance patterns of Gram-negative bacteria

	Value, N (%)													
**Antimicrobials**	*Acinetobacter baumannii/haemolyticus* (n= 17)	*Alcaligenes* sp. (n= 3)	*Citrobacter freundii* (n= 2)	*Enterobacter cloacae* (n= 6)	*Escherichia coli* (n= 37)	*Klebsiella ornithinolytica* (n= 1)	*Klebsiella pneumoniae* (n= 5)	*Morganella morganii* (n= 5)	*Proteus mirabilis* (n= 20)	*Proteus vulgaris* (n= 1)	*Providencia* sp. (n= 2)	*Pseudomonas aeruginosa* (n= 72)	*Pseudomonas fluorescens/ Putida* (n= 1)	*Serratia marcescens* (n= 7)
Trimethoprim/sulfamethoxazole	15 (88.2)	0 (0.0)	1 (50.0)	0 (0.0)	15 (40.6)	1 (100.0)	3 (60.0)	1 (20.0)	14 (70.0)	1 (100.0)	0 (0.0)	1 (1.4)	**-**	**-**
Piperacillin/tazobactam	-	0 (0.0)	1 (50.0)	1 (16.7)	3 (8.1)	0 (0.0)	2 (40.0)	1 (20.0)	0 (0.0)	0 (0.0)	0 (0.0)	13 (18.1)	0 (0.0)	0 (0.0)
Piperacillin	4 (23.5)	0 (0.0)	1 (50.0)	1 (16.7)	19 (51.3)	0 (0.0)	4 (80.0)	3 (60.0)	16 (80.0)	0 (0.0)	0 (0.0)	10 (13.9)	0 (0.0)	0 (0.0)
Meropenem	12 (70.6)	-	-	0 (0.0)	0 (0.0)	-	1 (20.0)	0 (0.0)	0 (0.0)	0 (0.0)	0 (0.0)	4 (5.6)	0 (0.0)	0 (0.0)
Levofloxacin	12 (70.6)	2 (66.7)	-	0 (0.0)	4 (11.0)	-	-	-	-	-	-	8 (11.1)	-	0 (0.0)
Gentamicin	12 (70.6)	2 (66.7)	1 (50.0)	1 (16.7)	4 (11.0)	0 (0.0)	2 (40.0)	1 (20.0)	12 (60.0)	0 (0.0)	0 (0.0)	9 (12.5)	.	0 (0.0)
Ceftazidime	-	0 (0.0)	0 (0.0)	0 (0.0)	0 (0.0)	-	-	0 (0.0)	-	-	-	8 (11.1)	0 (0.0)	-
Tobramycin	-	0 (0.0)	0 (0.0)	0 (0.0)	0 (0.0)	-	-	-	-	-	-	2 (2.8)	0 (0.0)	0 (0.0)
Ampicillin	5 (29.4)	-	-	6 (100.0)	22 (59.4)	1 (100.0)	4 (80.0)	3 (60.0)	15 (75.0)	1 (100.0)	0 (0.0)	-	-	5 (71.4)
Imipenem	-	-	-	0 (0.0)	0 (0.0)	0 (0.0)	0 (0.0)	0 (0.0)	0 (0.0)	0 (0.0)	0 (0.0)	6 (8.3)	0 (0.0)	0 (0.0)
Amikacin	12 (70.6)	0 (0.0)	0 (0.0)	0 (0.0)	0 (0.0)	0 (0.0)	1 (20.0)	0 (0.0)	1 (5.0)	0 (0.0)	0 (0.0)	7 (9.7)	0 (0.0)	0 (0.0)
Cefazolin	-	-	-	4 (66.7)	20 (54.1)	1 (100.0)	2 (40.0)	3 (60.0)	12 (60.0)	1 (100.0)	1 (50.0)	-	-	3 (42.8)
Colistin	0 (0.0)	-	-	-	0 (0.0)	-	-	-	-	-	-	-	-	-
Cefotaxime	-	0 (0.0)	0 (0.0)	2 (33.3)	9 (24.3)	1 (100.0)	4 (80.0)	0 (0.0)	13 (65.0)	0 (0.0)	0 (0.0)	-	-	-
Amoxicillin/clavulanic acid	-	-	-	6 (100.0)	6 (16.2)	0 (0.0)	2 (40.0)	3 (60.0)	5 (25.0)	1 (100.0)	1 (50.0)	-	-	5 (71.4)
Ciprofloxacin	-	-	-	0 (0.0)	8 (22.0)	-	-	-	-	-	-	28 (38.9)	0 (0.0)	1 (14.3)
Cefepime	-	2 (66.7)	-	-	-	-	-	0 (0.0)	-	-	-	9 (12.5)	0 (0.0)	-
Cefuroxime	-	-	-	-	0 (0.0)	-	1 (20.0)	-	-	-	-	-	-	-
Tetracycline	-	0 (0.0)	-	-	-	-	-	-	-	-	-	-	-	-
Fosfomycin	-	-	-	-	0 (0.0)	-	-	-	-	-	-	-	-	-
Aztreonam	-	2 (66.7)	-	0 (0.0)	-	-	-	-	-	-	-	6 (8.3)	-	-
Nitrofurantoin	-	-	-	-	0 (0.0)	-	-	-	-	-	-	-	-	-
Ertapenem	-	-	-	0 (0.0)	-	-	-	-	-	-	-	-	-	-

Table S2. Drug resistance patterns of Gram-positive bacteria

	Value, N (%)												
**Antimicrobials**	*Enterococcus avium* (n= 1)	*Enterococcus faecalis* (n= 3)	*Staphylococcus aureus* (n= 90)	*Staphylococcus auricularis* (n= 2)	*Staphylococcus epidermidis* (n= 3)	*Staphylococcus haemolyticus* (n= 5)	*Staphylococcus lugdunensis* (n= 2)	*Staphylococcus schleiferi ss, coagulans* (n= 1)	*Staphylococcus sciuri* (n=1)	*Staphylococcus simulans* (n= 2)	*Streptococcus agalactiae* (n= 1)	*Streptococcu s pyogens* (n= 1)	*Streptococcu s salivaris* (n= 1)
Trimethoprim/ sulfamethoxazole	-	-	8 (8.9)	-	0 (0.0)	2 (40.0)	0 (0.0)	0 (0.0)	0 (0.0)	0 (0.0)	0 (0.0)	0 (0.0)	-
Penicillin	-	-	59 (65.6)	1 (50.0)	3 (100.0)	-	1 (50.0)	0 (0.0)	-	2 (100.0)	0 (0.0)	0 (0.0)	0 (0.0)
Vancomycin	0 (0.0)	0 (0.0)	0 (0.0)	-	0 (0.0)	0 (0.0)	0 (0.0)	-	-	0 (0.0)	0 (0.0)	0 (0.0)	0 (0.0)
Teicoplanin	0 (0.0)	0 (0.0)	0 (0.0)	-	0 (0.0)	-	0 (0.0)	-	1 (100.0)	0 (0.0)	-	-	-
Oxacillin	-	-	19 (21.1)	1 (50.0)	2 (66.7)	2 (40.0)	0 (0.0)	0 (0.0)	1 (100.0)	1 (50.0)	-	-	-
Meropenem	-	-	-	-	-	-	-	-	-	-	-	-	0 (0.0)
Levofloxacin	-	-	-	-	-	1 ((20.0)	-	-	-	-	-	-	1 (100.0)
Linezolid	0 (0.0)	0 (0.0)	1 (1.1)	0 (0.0)	0 (0.0)	0 (0.0)	0 (0.0)	-	-	0 (0.0)	-	-	-
Gentamicin	-	-	25 (27.8)	0 (0.0)	2 (66.7)	3 (60.0)	1 (50.0)	0 (0.0)	1 (100.0)	1 (50.0)	-	-	-
Ampicillin	0 (0.0)	0 (0.0)	64 (71.1)	1 (50.0)	3 (100.0)	2 (40.0)	0 (0.0)	-	-	2 (100.0)	-	0 (0.0)	0 (0.0)
Rifampicin	-	-	6 (6.7)	1 (50.0)	1 (33.3)	0 (0.0)	-	-	-	-	-	-	-
Daptomycin	-	0 (0.0)	1 (1.1)	0 (0.0)	0 (0.0)	0 (0.0)	0 (0.0)	-	1 (100.0)	-	-	-	-
Cefotaxime	-	-	-	-	-	-	-	-	-	-	-	0 (0.0)	0 (0.0)
Amoxicillin/clav ulanic acid	0 (0.0)	0 (0.0)	10 (11.1)	1 (50.0)	2 (66.7)	2 (40.0)	-	-	-	0 (0.0)	-	-	-
Ciprofloxacin	-	-	13 (14.4)	-	-	0 (0.0)	-	-	-	0 (0.0)	-	-	-
Netilmicin	-	1	0 (0.0)	0 (0.0)	0 (0.0)	0 (0.0)	0 (0.0)	-	-	0 (0.0)	-	-	-
Erythromycin	-	-	21 (23.3)	1 (50.0)	3 (100.0)	4 ((80.0)	1 (50.0)	0 (0.0)	1 (100.0)	0 (0.0)	0 (0.0)	0 (0.0)	0 (0.0)
Clindamycin	-	-	14 (15.6)	-	-	1 (20.0)	-	0 (0.0)	-	0 (0.0)	0 (0.0)	-	0 (0.0)
Cefepime	-	-	-	-	-	-	-	-	-	-	-	0 (0.0)	-
Cefuroxime	-	-	-	-	-	-	-	-	-	-	-	0 (0.0)	1 (100.0)
Ceftriaxone	-	-	-	-	-	-	-	-	-	-	-	0 (0.0)	0 (0.0)
Cefaclor	-	-	-	-	-	-	-	-	-	-	-	0 (0.0)	1 (100.0)
Azithromycin	-	-	-	-	-	-	-	-	-	-	-	0 (0.0)	0 (0.0)
Fosfomycin	-	-	-	-	0 (0.0)	0 (0.0)	-	-	-	-	-	-	-
Clarithromycin	-	-	-	-	2 (66.7)	-	-	-	-	-	-	-	-
